# Night shift work and cardiovascular diseases among employees in Germany: five-year follow-up of the Gutenberg Health Study

**DOI:** 10.5271/sjweh.4139

**Published:** 2024-04-01

**Authors:** Sylvia Jankowiak, Karin Rossnagel, Juliane Bauer, Andreas Schulz, Falk Liebers, Ute Latza, Karla Romero Starke, Andreas Seidler, Matthias Nübling, Merle Riechmann-Wolf, Stephan Letzel, Philipp Wild, Natalie Arnold, Manfred Beutel, Norbert Pfeiffer, Karl Lackner, Thomas Münzel, Alicia Schulze, Janice Hegewald

**Affiliations:** a shared first authorship; 1 Division Work and Health, Federal Institute for Occupational Safety and Health (BAuA), Berlin, Germany.; 2 Preventive Cardiology and Preventive Medicine, Department of Cardiology, University Medical Center of the Johannes Gutenberg University Mainz, Mainz, Germany.; 3 Institute and Policlinic of Occupational and Social Medicine (IPAS), Faculty of Medicine Carl Gustav Carus, TU Dresden, Dresden, Germany.; 4 Institute of Sociology, Faculty of Behavioral and Social Sciences, TU Chemnitz, Chemnitz, Germany.; 5 FFAW: Freiburg Research Centre for Occupational Sciences, Freiburg, Germany.; 6 Institute of Occupational, Social, and Environmental Medicine, University Medical Center of the Johannes Gutenberg University Mainz, Mainz, Germany.; 7 Center for Thrombosis and Hemostasis (CTH), University Medical Center of the Johannes Gutenberg University Mainz, Mainz, Germany.; 8 German Center for Cardiovascular Research (DZHK), Partner Site Rhine-Main, Mainz, Germany.; 9 Institute of Molecular Biology (IMB), Mainz, Germany.; 10 Department of Cardiology, University Heart and Vascular Center Hamburg, Hamburg, Germany.; 11 German Center for Cardiovascular Research (DZHK), Partner Site Hamburg/Kiel/Luebeck, Hamburg, Germany.; 12 Department of Psychosomatic Medicine and Psychotherapy, University Medical Center of the Johannes Gutenberg University Mainz, Mainz, Germany.; 13 Department of Ophthalmology, University Medical Center of the Johannes Gutenberg University Mainz, Mainz, Germany.; 14 Institute for Clinical Chemistry and Laboratory Medicine, University Medical Center of the Johannes Gutenberg University Mainz, Mainz, Germany.; 15 Department of Cardiology – Cardiology I, University Medical Center of the Johannes Gutenberg University Mainz, Mainz, Germany.; 16 Institute of Medical Biostatistics, Epidemiology and Informatics (IMBEI), University Medical Center of the Johannes Gutenberg University Mainz, Mainz, Germany.

**Keywords:** cohort study, CVD, GHS, occupational, Rhineland-Palatinate, workplace

## Abstract

**Objective:**

This study aimed to determine if there is an increased risk of incident cardiovascular diseases (CVD) resulting from cumulative night shift work in the German population-based Gutenberg Health Study (GHS).

**Methods:**

We examined working participants of the GHS at baseline and after five years. Cumulative night shift work in the 10 years before baseline was assessed and categorized as low (1–220 nights ≙ up to 1 year), middle (221–660 nights ≙ 1–3 years), and high (>660 nights ≙ 3 years) night shift exposure. Hazard ratios (HR) were estimated for incident “quality-assured CVD events” using Cox proportional hazard models.

**Results:**

At baseline, 1092 of 8167 working participants performed night shift work. During the follow-up, 202 incident cardiovascular events occurred. The crude incidence rates for CVD per 1 000 person-years were 6.88 [95% confidence interval (CI) 4.80–9.55] for night shift workers and 5.19 (95% CI 4.44–6.04) for day workers. Cumulative incidence curves showed a higher cumulative incidence in workers exposed to night shift work compared to day workers after five years. The adjusted HR for incident CVD events were 1.26 (95% CI 0.68–2.33), 1.37 (95% CI 0.74–2.53)and 1.19 (95% CI 0.67–2.12) for employees in the low, middle and high night shift categories compared to employees without night shift work, respectively.

**Conclusions:**

The observed tendencies indicate that night shift work might be negatively associated with cardiovascular health. We expect the continued follow-up will clarify the long-term impact of night shift work.

Night shift work affects 15–20% of the working population in Europe, a figure that has not changed much since 2005 (1). These employees work at night to meet the interests and demands of many manufacturing, healthcare, and service industries in our 24/7 society. Shift work and the associated circadian disruption can influence the risk of cardiovascular disease in several interacting ways. One consequence is sleep deprivation and poor sleep quality (2). In addition, the limited time available for rest and participation in activities can lead to social isolation and stress (3, 4). A third pathway is lifestyle changes associated with shift work (eg, dietary habits, smoking, alcohol consumption, etc.) (5). This can lead to inflammatory changes, changes in blood coagulation, activation of the sympathetic nervous system, and an increase in blood pressure, contributing to an increased risk of cardiovascular disease (6, 7).

In a previous cross-sectional study, we examined the association between current and cumulative exposure to night shift work and measures of arterial stiffness, vascular function, and intima-media thickness (IMT) (8). We found arterial stiffness increased and vascular function, decreased, whereas IMT did not change with increasing exposure to night shift work. These associations were reduced after adjusting for lifestyle risk factors. This supports previous research finding negative associations between night shift work and endothelial function (9–11), and also suggest a potential pathway involving lifestyle changes.

Research suggests an association between cardiovascular diseases (CVD) and night shift work. A recent umbrella review of eight systematic reviews and meta-analyses, examined associations of night shift work with various health outcomes. One of the strongest associations (‘highly suggestive evidence’) was observed between ever having worked shifts, including night shifts, (compared with never having worked shifts) and myocardial infarction (MI) (12). Several other systematic reviews, such as that of Torquati et al (4) showed that shift workers (with night work in the majority of studies) had an approximately 20% higher risk of mortality from CVD [1.22, 95% confidence interval (CI) 1.09–1.37] and coronary heart disease (CHD) (1.18, 95% CI 1.06–1.32) compared with non-shift workers. The risk of any CVD event was 17% higher in shift workers than in day workers, and the risk of CHD morbidity was 26% higher (1.26, 95% CI 1.10–1.43). Another meta-analysis by Su et al (13) came to similar conclusions: compared with regular day workers, the pooled risk of cardiovascular mortality was 1.15 (95% CI 1.03–1.29) for those ever exposed to shift work. The association with ischemic heart disease (IHD) was analyzed in another meta-analysis (14). The pooled risk for the association between night shift work and the risk of IHD was 1.44 (95% CI 1.10–1.89). Further evaluation of the dose–response relationship showed that each one-year increase in shift work was associated with a risk ratio (RR) of 1.009 (95% CI 1.006–1.012). However, another umbrella review, this time including all types of studies, found only low-grade evidence of an association between shift work and IHD, MI, and ischemic stroke (15).

The present study aims to investigate the effect of cumulative night shift work during the ten years before baseline and the incidence of cardiovascular disease during a five-year follow-up using data from the Gutenberg Health Study (GHS) cohort. As studies reporting risk estimates based on the duration of exposure to shift work and considering potential mediators between shift work and health outcomes are scarce (4), we endeavored to improve on previous research by using a more precise estimate of cumulative night shift exposure based on the number of nights worked per month over the last ten years of work. This cumulative measure is a summation of all the nights worked in the ten years prior to baseline. We also consider potential mediators to examine their impact on any associations between night shift work and cardiovascular risk.

## Methods

The GHS is a population-based cohort study that recruited a random sample of residents aged 35–74 years and living in the City of Mainz and Mainz-Bingen district (Rhineland-Palatinate, Germany) starting in 2007. The cohort initially focused on cardiovascular health and its preclinical markers; numerous other health outcomes were included in a later stage.

At baseline, a total of N=15 010 participants was recruited with a response of 60.0% (16). Baseline assessments included social, lifestyle, and occupation factors, as well as cardiovascular health and function examination in a five-hour examination conducted at the University Medical Centre in Mainz, Germany, between April 2007 and April 2012. For follow-up, the GHS study examined each participant at the same time of year and a similar time of day, including various interviews, blood sampling, and clinical examinations (17). Of the 15 010 initial participants, 13 417 participated at the follow-up five years later between April 2012 to April 2017 (retention rate 91.6%).

**Figure 1:**
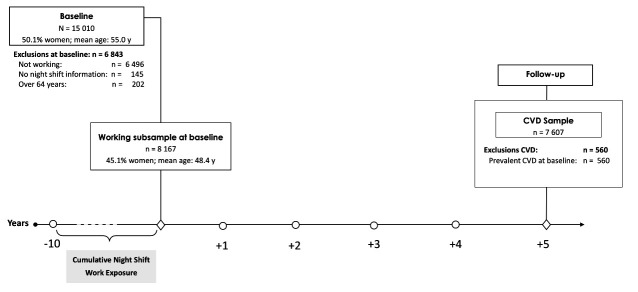
Flow diagram of participation (analysis sample) and timeline.

Cardiovascular effects of night shift work were examined with a subsample of 8167 participants working at baseline. Individuals were excluded from analyses if they were older than 64 years, not in paid employment, or did not provide any occupational information. Also, participants with CVD outcomes at or before baseline were excluded (560 participants), resulting in the analyzed sample of 7607 workers. A study flowchart depicting the subsamples of participants included in the analyses and the number of persons excluded or lost to follow-up is shown in figure 1. The study is a mixture of retrospectively recorded night shift work (ten years before baseline) and prospectively examined incident cardiovascular events (five years after baseline).

### Variables

*Exposure to cumulative night shifts.* At baseline, subjects were requested to describe past occupational periods and their current job. A maximum of 15 occupational periods were covered within an interview, and each job was subsequently coded using the current German classification of occupations KldB 2010 (5 digits) (18). On average, participants reported four periods. The number of night shifts per month was reported for each occupational period. We defined a night shift as any work during 23:00–05:00 hours and assessed this with the question, “Did you work between the hours of 23:00 and 05:00 at this job?”

Cumulative night shifts were calculated retrospectively for ten years before baseline. For each occupational period within the last ten years, the reported number of night shifts per month was multiplied by 11 months (since approximately one month of annual leave is standard in Germany) and then multiplied by the number of years in that period. Subsequently, the number of night shifts worked were summed for each participant and categorized as ‘no exposure’ = 0 nights, ‘low exposure’ = 1–220 nights, ‘medium exposure’ = 221–660 nights, and ‘high exposure’ = >660 nights (analogous to our baseline publication) (8). The cut-off value of 220 nights equals the accumulation of one work year in night shift work (ie, 250 annual working days minus 30 days of annual leave).

### Cardiovascular outcomes

Incident CVD were defined as a first acute MI (ICD-10: I21), cerebral infarction/stroke (ICD-10: I63), coronary artery disease (ICD-10: I25.1), atrial fibrillation (ICD-10: I48) or confirmed sudden cardiac deaths (ICD-10: I46) occurring during the follow-up period. All the diseases above were analyzed together.

Medical records, as well as information from physicians and from the study participants themselves, were collected and submitted to a team of experts In the case of death, the death certificate was obtained from the public health authorities. The team of experts was composed of two physicians and an epidemiologist and evaluated the documents in regular meetings. The team reviewed the information to identify diseases relevant to the study (17).

### Covariates

We considered age, sex, socioeconomic status (SES), smoking, alcohol intake, and the occupational variables being a manager/supervisor, job complexity level, overtime per week, and occupational noise from interviews at baseline. Additionally, we considered the dispositional variables waist-to-height ratio (WHtR), menopause status, and family history of MI or stroke as potential confounders from clinical examinations at baseline.

Age was categorized into decades. SES was assessed by an index score comprising school and professional education, occupational position, and salary (19, 20). Smoking was dichotomized into current/occasional smokers and never/ex-smokers considering the last 12 months. Alcohol categories were built according to the sex-specific categories “above tolerable limit” (>10–40g/day for women and >20–60g/day for men) and “abuse” (>40g/day for women and >60g/day for men), as recommended in Germany.

Information on occupational factors were collected during the assessment of occupational periods. The KldB 2010 is coded in five digits and the dichotomous variable of being in a management position was obtained from the fourth digit of the occupational code. Job complexity is obtained from the last digit and contains four levels: low (helpers), medium (skilled workers), complex (specialists) and very complex (experts). Contractual working time and overtime per week were assessed by the question: “On average, how many hours per week were you working during a) fixed working hours and b) overtime?” and was used continuously in hours. Participants were also asked about their exposure to occupational noise and asked to describe the noise exposure by selecting a comparable source of noise (1=refrigerator, …, 5=pneumatic hammer).

A numerical index of body proportion, WHtR was chosen as it is a better discriminator of cardiovascular risk factors than body mass index (BMI) (21). WHtR is defined as waist circumference divided by height and used continuously and per standard deviation (SD). Menopausal status was requested from women with the question if their menstruation was still regular. A positive family history regarding cardiovascular events was assumed if MI or stroke were reported for first-degree relative females ≤65 years or males ≤60 years.

### Statistical analysis

Characteristics of the study population and the subclinical parameters were described using averages, SD, medians, and quartiles for continuous variables or absolute frequencies (N) and percentages (%) for categorical variables. Descriptive analyses were carried out for the sample stratified by night shift work in three categories: 1–220 nights (low exposure), 221–660 nights (middle exposure) and >660 nights (high exposure) within the last decade before baseline and for the sample without night shift work.

Since individuals were observed for different lengths of time (some completed the baseline questionnaire earlier than others), the individual person-time at risk was calculated and taken into account in the regression analyses. Time-to-event was defined by time (years and months) from baseline to the first occurrence of a CVD event. Participants who discontinued the study participation not related to CVD were right censored. Cumulative incidence curves were used to graphically depict the differences in CVD incidence between exposure groups.

To examine the association of night shift work ten years before baseline and incident CVD in the follow-up period, we estimated hazard ratios (HR) using Cox regression. Using a method described previously (8), we selected three adjustment models for Cox regressions: (i) model 1: a basic model adjusted for age and sex; (ii) model 2: model 1 and occupational variables: being a manager/supervisor, job complexity, overtime work, and occupational noise; and (iii) model 3: model 2 and lifestyle factors: smoking, alcohol intake, and SES and the dispositional variables: WHtR, menopausal status, family history of MI or stroke.

In addition, all regression models were stratified by sex.

We focus on the results of model 2, as the subsequent model includes potential intermediate risk factors (WHtR, smoking, alcohol intake) and dispositional risk factors (family history of MI or stroke). Thus, model 3 is a better depiction of the direct effect night shift exposure on CVD risk while model 2 should give an indication of the total effect. All analyses were conducted using R version 4.1.0 (22).

## Results

At baseline, 1092 of the 8167 employees (13.4%) had ever worked night shifts (7.6% and 18.1% of women and men, respectively). Although men made up slightly more than half of the population (55%), they were much more likely to be exposed to night shifts. Men comprised around two-thirds of those with a low exposure of 1–220 night shifts (67.8%) and a middle exposure of 221–660 night shifts (67.8%). In the high-exposure category of >660 night shifts, the proportion of men rose to 77.7% (table 1). Day workers were, on average, about two years older than workers in the low- and middle-cumulative night shift exposure categories. Employees with more than 660 night shifts differed from the other subgroups in various characteristics: the high exposure night shift group had the lowest SES, and the highest share of full-time employees, and they experienced more noise at work. Employees in the high exposure night shift group had the longest average job retention period, were less likely to work overtime, and had the lowest proportion of people drinking above tolerable alcohol levels. The distribution of night shift work across occupational groups is shown in the supplementary material (www.sjweh.fi/article/4139, table A).

**Table 1:** Population characteristics at baseline [MI=myocardial infarction; SD=standard deviation; SES=socioeconomic status; WHtR=waist-to-height ratio]

		Total			No night shift work			1–220 nights Median=1 night/ month (0/2)			221–660 nights Median=4 nights/month (1/5)			>660 nights Median=9 nights/ month (7/15)	
		% (N)	Mean (SD)		% (N)	Mean (SD)		% (N)	Mean (SD)		% (N)	Mean (SD)		% (N)	Mean (SD)
Total		8167			7027			397			366			377	
Sex															
	Women	45.1 (3684)			47.7 (3354)			32.2 (128)			32.2 (118)			22.3 (84)	
	Men	54.9 (4483)			52.3 (3673)			67.8 (269)			67.8 (248)			77.7 (293)	
Age (years)			48.4 (7.6)			48.6 (7.6)			46.5 (7.5)			46.4 (7.3)			48.0 (7.4)
SES (3–21)			14.11 (4.23)			14.18 (4.22)			14.56 (4.24)			14.35 (4.23)			11.93 (3.78)
Smoking		23.8 (1947)			22.8 (1603)			31.5 (125)			27.9 (102)			31.0 (117)	
Alcohol above tolerable limit ^a^		23.7 (1933)			24.3 (1710)			23.4 (93)			20.2 (74)			14.9 (56)	
Alcohol abuse ^a^		2.6 (211)			2.6 (180)			3.5 (14)			2.7 (10)			1.9 (7)	
Job complexity															
	Low	3.6 (292)			3.6 (252)			3.3 (13)			3.0 (11)			4.2 (16)	
	Medium	44.7 (3651)			44.5 (3130)			34.5 (137)			40.7 (149)			62.3 (235)	
	High	20.9 (1705)			20.5 (1437)			24.2 (96)			26.0 (95)			20.4 (77)	
	Very high	30.8 (2518)			31.4 (2207)			38.0 (151)			30.3 (111)			13 (49)	
Management Position		15.5 (1268)			15.6 (1095)			18.6 (74)			12.8 (47)			13.8 (52)	
Overtime per week [hours]			3.48 (5.83)			3.34 (5.62)			4.72 (6.67)			4.81 (7.74)			3.38 (6.33)
Full time work		77.6 (6341)			75.9 (5331)			88.2 (350)			86.9 (318)			90.7 (342)	
Noise at work		24.7 (2013)			22.2 (1561)			35.0 (139)			36.1 (132)			48.0 (181)	
Years at current workplace			14.2 (10.5)			14.4 (10.6)			9.9 (8.5)			11.5 (9.4)			15.9 (10.4)
WHtR			0.54 (0.08)			0.54 (0.08)			0.54 (0.07)			0.54 (0.07)			0.56 (0.08)
Family history of MI or stroke		32.2 (2632)			32.3 (2268)			33.5 (133)			29.0 (106)			33.2 (125)	
Still regular menstruation		50.3 (1852)			49.5 (1660)			64.8 (83)			54.2 (64)			53.6 (45)	

^a^ Alcohol limits for men: above tolerable limit >20–60 g/day; abuse >60g/day; limits for women: above tolerable limit >10–40; abuse >40g/day

Altogether, 7607 participants were analyzed for CVD incidence. During the five-year follow-up, 202 incident cardiovascular events occurred, 167 among non-night shift and 35 among night shift workers (at baseline). Of the 202 incident cardiovascular events, 11 were cardiac-related deaths, including but not limited to sudden cardiac deaths. The incidence rate (IR) per 1 000 person-years was 6.88 (95% CI 4.80–9.55) for night shift workers and 5.19 (95% CI 4.44–6.04) for day workers (see table 2). Three quarters (N=156) of the 202 incident cardiovascular events were registered for men. Among men, the incidence rate per 1000 person-years was higher for night shift workers compared to day workers: IR 8.66 (95% CI 5.89–12.28) vs. IR 7.63 (95% CI 6.35–9.08). In women, the incidence rate per 1000 person-years was at the same level for night shift workers and for female day workers: IR 2.65 (95% CI 0.72–6.77) vs. 2.66 (95% CI 1.92–3.60), respectively. There were only four CVD events in female night shift workers, reflected in the broad CI.

**Table 2:** Five-year incidence rates for people with or without a history of night shift work (free of CVD) in the working GHS population. [CI=confidence interval; PY=person years.]

		N participants	N events	Censored events	PY	Crude incidence rate per 1000 PY (95% CI)
Total		7607	202	7443	37 256	5.42 (4.70–6.22)
	No night shift work	6561	167	6425	32 169	5.19 (4.44–6.04)
	Night shift work	1046	35	1018	5088	6.88 (4.80–9.55)
Men		4103	156	3973	19 964	7.81 (6.64–9.13)
	No night shift work	3364	125	3261	16 386	7.63 (6.35–9.08)
	Night shift work	739	31	712	3578	8.66 (5.89–12.28)
Women		3504	46	3470	17 292	2.66 (1.95–3.55)
	No night shift work	3197	42	3164	15 783	2.66 (1.92–3.60)
	Night shift work	307	4	306	1510	2.65 (0.72–6.77)

**Figure 2:**
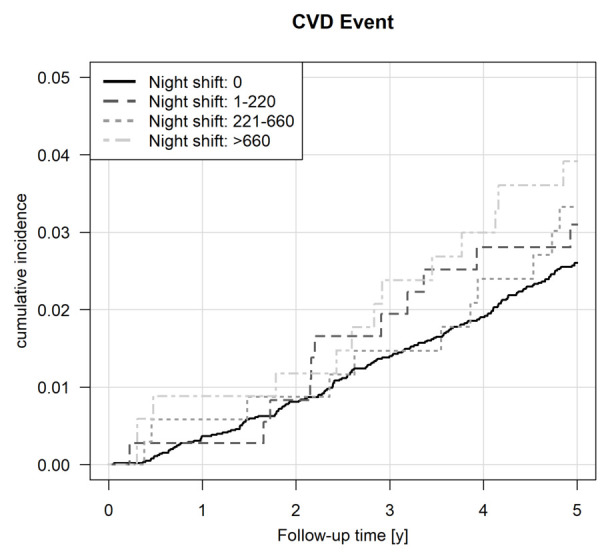
Survival analyses based on the number of night shifts during the 10 years before baseline: Incidence plot for cardiovascular diseases (CVD), ie, incident myocardial infarction (ICD-10: I21), cerebral infarction/stroke (ICD-10: I63), coronary artery disease (ICD-10: I25.1), atrial fibrillation (ICD-10: I48) or confirmed sudden cardiac death (ICD-10: I46) occurring during the follow-up period.

Cumulative incidence curves for the nightshift exposure categories are shown in figure 2. Based on these unadjusted curves, the cumulative incidence levels at five years were higher in the group of workers with the highest cumulative nightshift exposure compared with the unexposed group. Throughout the follow-up period, the cumulative incidence was generally higher in the nightshift-exposed groups than in the unexposed group.

Table 3 shows the CVD risks for participants with varying levels of prior accumulated night shift exposure compared to non-night shift workers (N=7587 due to 20 missing covariates). The Cox regression analyses revealed unadjusted HRs of 1.20 (95% CI 0.65–2.20), 1.27 (95% CI 0.69–2.34), and 1.52 (95% CI 0.86–2.67) for the low-, middle- and high-exposure categories. For the whole sample, all adjusted models showed that the middle exposure category with 1 to 3 years of accumulated night shift work revealed the highest risk estimates for CVD (HR 1.27–1.37), followed by the high exposure category of >3 cumulative years in night shift work (HR 1.14–1.52). Nevertheless, none of the models gained statistical significance. Stratified by sex, men reflected the same picture as the whole sample in model 2 (HR 1.43, 95% CI 0.75–2,73). In contrast, women showed the highest CVD risks in the low night shift work category (HR 1.75, 95% CI 0.41–7.31) but the CI for women were very wide.

**Table 3:** Hazard ratios (HR) and 95% confidence intervals (CI) from Cox regression models for incident cardiovascular disease (CVD) according to cumulative night shift work in the 10 years prior to baseline in low, middle and high exposure categories compared to no night shift work.

		No night shift work			1–220 nights			221–660 nights			>660 nights	
		N events	HR (95% CI)		N event	HR (95% CI)		N event	HR (95% CI)		N event	HR (95% CI)
**Total (N=7587 people, 202 events)** ^a^		167			11			11			13	
	Model 0		1 (Ref.)			1.20 (0.65–2.20)			1.27 (0.69–2.34)			1.52 (0.86–2.67)
	Model 1 ^b^					1.21 (0.66–2.23)			1.35 (0.73–2.50)			1.32 (0.75–2.32)
	Model 2 ^c^					1.26 (0.68–2.33)			1.37 (0.74–2.53)			1.19 (0.67–2.12)
	Model 3 ^d^					1.19 (0.64–2.21)			1.32 (0.71–2.44)			1.14 (0.64–2.04)
**Men (N=4098 people, 156 events) ^e^**		125			9			10			12	
	Model 0		1 (Ref.)			0.99 (0.50–1.95)			1.18 (0.62–2.26)			1.24 (0.69–2.24)
	Model 1 ^b^					1.14 (0.58–2.24)			1.43 (0.75–2.73)			1.35 (0.74–2.44)
	Model 2 ^c^					1.18 (0.60–2.33)			1.43 (0.75–2.73)			1.20 (0.70–2.19)
	Model 3 ^d^					1.11 (0.56–2.19)			1.37 (0.72–2.63)			1.15 (0.63–2.10)
**Women (N=3489 people, 46 events) ^e^**		42			2			1			1	
	Model 0		1 (Ref.)			1.30 (0.31–5.36)			0.68 (0.09–4.91)			0.99 (0.14–7.20)
	Model 1					1.70 (0.41–7.06)			0.87 (0.12–6.31)			1.04 (0.14–7.54)
	Model 2					1.75 (0.42–7.31)			0.93 (0.13–6.89)			1.00 (0.13–7.06)
	Model 3					1.72 (0.50–7.20)			0.89 (0.12–6.63)			0.91 (0.12–6.75)

^a^ Missings covariates (N=20) ^b^ Adjusted for age & sex; *adjusted for age ^c^ Model 1 + adjusted for occupational variables (management/supervisor position, job complexity, overtime per week, noise) ^d^ Model 2 + adjusted for SES, smoking status, alcohol above tolerable limit, WHtR (per SD), menopausal status, family history of MI or stroke

## Discussion

In this study, we used interview data about working hours ten years before baseline to examine if cumulative night shift work increases the risk of CVD five years later in a cohort study of 8167 employees in Rhineland-Palatinate, Germany. The survival analyses descriptively showed that after five years, employees in the high night shift work category had an earlier onset of CVD up to one and a half years compared to employees who worked during the day. The unadjusted IR was higher for night shift workers than day workers. Still, using a Cox proportional hazard model and adjusting for covariates, we found only statistically non-significant increased risks for incident CVD according to night shift work.

Analyses examining sex differences generally found that the global effects were similar for men, reflecting the more than two times higher share of men in night shift work. In contrast, the picture for women differed concerning CVD. Women showed the highest CVD risks in the low night shift work category, indicating that women’s risk surpasses the risk of men in that category. However, female participants had far fewer incident cardiovascular events than men (46 vs. 156 events).

### Comparison to other studies

Past systematic reviews and meta-analyses suggest an increased risk of CVD from night shift work (4, 12, 14, 23–25). Vyas et al (25) observed associations between shift work and MI (RR 1.23, 95% CI 1.15–1.31), ischemic stroke (RR 1.05, 95% CI 1.01–1.09), and coronary events (RR 1.24, 95% CI 1.10–1.39). Torquati et al (4) showed that the association between shift work and CVD was non-linear with a first appearance after five cumulative years in shift work and a 7.1% increase in risk of CVD events for every additional five years of exposure (95% CI 1.05–1.10).

A new prospective cohort study including 238 661 participants from the UK Biobank reported a higher risk of incident (HR 1.11, 95% CI 1.06–1.19) and fatal CVD (HR 1.25, 95% CI 1.08–1.44) (5). Kader et al (26) observed an increased risk of IHD among Swedish healthcare employees with permanent night shifts (HR 1.61, 95% CI 1.06–2.43) and night shifts >120 times per year (HR 1.53, 95% CI 1.05–2.21). Our results support those of previous studies. Although the effect estimates were not statistically significant, we observed an increased CVD with night shift work, especially in the medium and high exposure categories.

Analyses by Ho et al (5) using data from the huge UK Biobank showed that shift work was more strongly associated with cardiovascular events in women than in men (HR 1.16, 95% CI 1.07–1.27 vs. HR 1.08, 95% CI 1.02–1.14). A further prospective cohort study, including 189 158 female nurses, observed a statistically higher risk but slight absolute increase for coronary heart disease with increasing years of rotating night shift work (27). A study investigating the effects of night shift work on incident cerebrovascular disease observed an increased risk among employees over five years of night shift work (HR 1.87, 95% CI 1.27–2.77) (23).

In our study, there were only four CVD events among women with cumulative night shift experience and two CVD events among women working in night shift work at baseline during the five-year follow-up. This small number of events is extremely difficult to discuss and is unlikely to yield statistically significant results.

### Strengths and limitations

The main strength of this study is its prospective nature. In addition, CVD outcomes were measured objectively based on medical records and death certificates. Each CVD case were checked and certificated by an internal expert committee. Participants who did not appear for the follow-up examination but from whom death records, hospital discharge reports, or doctor’s letters with information on CVD were available were also included in the study. Various important individual and work-specific risk factors for CVD were collected and used in the analyses, eg, smoking status and long working hours. This is also an advantage, as previous studies have indicated a lack of studies considering lifestyle factors (4). Due to the standardized recruitment process over the entire recruitment period, the study population covered a large range of jobs.

The reported results should be considered against the background of their limitations. In general, it is possible that changes due to (latent) health problems were made already before the baseline assessment (“drift”) or because of the baseline assessment. In accordance with the German Working Hours Act (ArbZG), night workers were entitled to an occupational health examination prior to commencing night work and at regular intervals thereafter. These occupational medical examinations are offered every three years and annually upon reaching the age of 50 (ArbZG § 6). On the basis of these examinations, the occupational physician may recommend switching day shift work if there are indications of poor health. This could have led to improved health behaviors, reduced exposure to night shift work, and thus distorted association measurements.

Healthy worker bias is minimized since incident cases were included in the analyses even if workers left the workforce during the follow-up. Apart from that, a selection into night shift work of employees with unfavorable risk factor profiles can take place (28). The adjustment for risk factors should mitigate confounding due to this self-selection. Although we had a large sample, we examined a relatively short follow-up time of five years. In these five years, there were relatively few incident CVD, reflecting how healthier the GHS sample population is compared to other population samples in Germany (29). Male participants in the GHS aged 35–44 years and 45–54 years had a lower prevalence of hypertension (10.2% and 20.1%, respectively) compared to other German population-based studies, like SHIP-TREND (12.3% and 24.4%, respectively) and DEGS1 (12.8% and 23.1%, respectively) (29). Generally, events for CVD would have to be more frequent to detect significant results in time-to-event analyses. This lack of statistical power especially impacted the sex-stratified analyses. Women were less likely to be exposed to both higher cumulative levels of night work (or night shift work in general), and had fewer cases during follow-up, it is difficult to draw conclusions about any cardiovascular risk associated with night shift work in women. Other analyses of CVD from the five-year follow-up of the GHS, such as mobbing (30), work-life balance (31) and long working hours (32) also observed no statistically significant increases in CVD risks.

The study is based on a regional sample from the Mainz-Bingen area in Rhineland-Palatinate, Germany and may not be directly representative for other countries or other regions in Germany. When assessing the external validity or generalizability of the study, it must also be considered that persons with severe health restrictions and persons without sufficient knowledge of German were fundamentally excluded from participation in the study.

It has to be mentioned that “night shift work” can be carried out differently, eg, as a rotating or permanent night shift. One limitation of our study is that we do not have information on the form of shift rotation or permanent night shift work. However, permanent night shift work is rare in Germany (33). In the European Union a ‘night worker’ is defined as ‘any worker who, during night time, works at least three hours of his daily working time as a normal course’ (Article 2 (4)(a) of Directive 2003/88/EC). Each country is allowed to modify the European Regulation. The German Working Hours Act (ArbZG § 2) defines >2 hours of working between 23:00 –06:00 hours (22:00–05:00 for bakeries) as night work. We used a distinct description of exposure concerning the hours between 23:00–05:00. Yet, it may give rise to misclassification since employees with an evening shift until 24:00 were categorized as night shift workers.

We did not take changes in employment or the number of night shifts in the last five years into account. While night shift exposure during the five years between baseline and follow-up could have added to the impact of night shift work on cardiovascular risk, this ensured a comparable exposure assessment for all participants. Our exposure assessment was survey-based and updated at follow-up. Therefore, only the workers who survived to follow-up would have their exposure assessment updated. Like most population-based cohort studies on shift work, self-reported information on occupational exposure without validation by register data was used. The retrospectively self-reported night shift work over the last ten years may have led to recall bias and, thus, to non-differential misclassification bias. However, a study on the validity of the self-reported assessment of shift work showed a high sensitivity (96% and 90%) and specificity (92% and 96%) for questions about shift work with night shifts and permanent night shift (34).

### Concluding remarks

Our results suggest that night shift work, especially at high exposure levels, may be negatively associated with cardiovascular health. In this cohort study, we found an increased five-year risk of CVD between long-term night shift workers at baseline and CVD incidence after five years, but this relationship was not statistically significant. Thus, our study supports existing findings but with a high degree of uncertainty. Therefore, health promotion and monitoring of night shift workers should also aim to detect subclinical cardiovascular changes to prevent the development of cardiovascular diseases.

Further investigations and longitudinal data over a more extended period are needed to clarify the long-term effects of cumulative night shift work on cardiovascular health. Data from future follow-ups of the GHS should improve the study power and provide further conclusions.

## Supplementary material

Supplementary material

## Protection of research participants

Written informed consent was obtained from all participants. The local ethics committee and the local and federal data safety commissioners approved the study (#837.020.07(5555)).
