# Supplementary material for: Night shift work and cardiovascular diseases among employees in Germany: five-year follow-up of the Gutenberg Health Study
Source: Scand J Work Environ Health. 2024 Mar 27;50(3):142–51. doi: 10.5271/sjweh.4139 (PMC11006091; doi:10.5271/sjweh.4139)
Supplement: Supplementary material [file SJWEH-50-142-S001.pdf]

Night shift work and cardiovascular diseases among employees in Germany: five-year follow-up of the Gutenberg Health Study[1]

by Sylvia Jankowiak, MSc,[2] Karin Rossnagel, PhD, Juliane Bauer, MSc, Andreas Schulz, PhD, Falk Liebers, MD, Ute Latza, PhD, Karla Romero Starke, PhD, Andreas Seidler, MD, Matthias Nübling, PhD, Merle Riechmann-Wolf, MSc, Stephan Letzel, MD, Philipp Wild, MD, Natalie Arnold, MD, Manfred Beutel, MD, Norbert Pfeiffer, MD, Karl Lackner, MD, Thomas Münzel, MD, Alicia Schulze, PhD, Janice Hegewald, PhD

1. Supplementary material
2. Correspondence to: Sylvia Jankowiak, Division Work and Health, Federal Institute for Occupational Safety and Health (BAuA), Nöldnerstraße 40/42, 10317 Berlin. [E-Mail: jankowiak.sylvia@baua.bund.de]

Table S1. Subjects in occupational groups (numbers and column percentage)

	**Total**	**No night shift work**	**1–220 nights Median = 1 night/month (0/2)**	**221–660 nights Median = 4 nights/month (1/5)**	**>660 nights Median = 9 nights/month (7/15)**
	**% (n)**	**% (n)**	**% (n)**	**% (n)**	**% (n)**
**Total**	8 167	7 027	397	366	377
**Construction, architecture, surveying and building technology**	4.8 (395)	5.0 (348)	5.0 (20)	5.2 (19)	2.1 (8)
**Health, social affairs, teaching and Education**	19.1 (1 561)	18.7 (1 313)	22.9 (91)	27.9 (102)	14.6 (55)
**Commercial services, goods trading, sales, hotel and tourism**	9.8 (804)	10.1 (712)	9.6 (38)	4.6 (17)	9.8 (37)
**Agriculture, forestry, animal husbandry and horticulture**	2.8 (229)	2.9 (205)	3.3 (13)	1.9 (7)	1.1 (4)
**Natural Science, geography and computer science**	6.8 (555)	6.7 (473)	9.1 (36)	4.6 (17)	7.7 (29)
**Raw material extraction, production and manufacturing**	13.5 (1 102)	13.0 (911)	13.9 (55)	14.5 (53)	22.0 (83)
**Linguistics, literature, humanities, social and economic sciences, media, art, culture and design**	4.8 (394)	4.7 (333)	7.3 (29)	6.3 (23)	2.4 (9)
**Business organization, accounting, law and administration**	29.5 (2 407)	32.5 (2 283)	15.4 (61)	11.5 (42)	5.6 (21)
**Transport, logistics, protection and safety**	8.8 (719)	6.4 (448)	13.6 (54)	23.5 (86)	34.7 (131)
